# Effects of a Menstrual Health Education Intervention on Female Athletes’ Knowledge and Communication

**DOI:** 10.3390/sports14070266

**Published:** 2026-06-26

**Authors:** Mikaeli Carmichael, Alexandra Roberts, Kate Perry, Anthea Clarke

**Affiliations:** 1 Sport and Exercise Science, School of Allied Health, Human Services, and Sport La Trobe University, Melbourne, VIC 3086, Australia; 2 Coaching and Talent, Queensland Academy of Sport, Brisbane, QLD 4111, Australia

**Keywords:** health literacy, menstrual cycle, women, menstruation, monitoring

## Abstract

This study aims to determine the effect of an education intervention on female athletes’ menstrual health knowledge, communication, and perceived importance of menstrual cycle tracking, and whether factors such as age, education level, or hormonal contraceptive use influence these outcomes. Three athlete cohorts engaged in two workshops and received targeted handouts. Participants (n = 51) completed surveys before, after, and 3 months following the intervention, which included a menstrual health knowledge assessment and a series of ratings to ascertain perceived knowledge; likelihood that they would discuss menstrual health with teammates, coaches, medical staff, and performance staff; and perceived importance of menstrual cycle tracking. Bayesian generalised linear mixed models and Bayesian linear mixed models were used to understand the effect of time and interactions between time and age, educational level, or hormonal contraceptive use. Actual and perceived knowledge and likelihood to communicate with teammates and performance staff appeared to be higher post-intervention and were retained for at least three months. The importance of menstrual cycle tracking increased from post-intervention to follow-up, while no change in communication with coaches or medical staff was observed. There appeared to be some effect of age, education level, and hormonal contraceptive use on knowledge and communication with medical staff. A menstrual health education intervention could be a practical and effective strategy to promote menstrual health literacy and communication in sport settings.

## 1. Introduction

Menstrual cycle (MC) tracking is a widely discussed practice that aims to take female athletes’ physiology and experiences into account. There are many examples of high-profile athletes and sport organisations engaging in menstrual cycle tracking [1,2,3], but it does not appear to be widely implemented [4]. Social and context-specific barriers, such as stigma and a lack of understanding or value placed on menstrual health (MH), and athletes’ perception of practitioners’ competence, can influence the uptake of MC tracking [5]. Hence, the common recommendation in the literature is to provide education and promote open communication when implementing MC tracking in applied settings [6,7,8]. Education may encourage tracking by rationalising its use and conveying important information on how tracking data will be used, helping to promote buy-in and facilitate the informed consent process [9], and can provide athletes with the knowledge to proactively manage their health and performance [10,11]. Similarly, open communication during MC tracking implementation enables athletes and practitioners to collaborate and comfortably share personal or general information relating to an athlete’s menstrual health [12].

Previous research demonstrates athletes generally have poor MH literacy, which has also led to recommendations for more MH education strategies to be introduced in sport settings [13,14,15,16]. Most articles examining the effect of female-specific education interventions with athletes tend to focus on knowledge of the female athlete triad (FT) or relative energy deficiency in sport (REDs) [17,18,19,20], which are only some topics relevant to MH for athletes. To date, there are few studies on interventions that address knowledge of other or broader MH topics [15,16]; however, they do support the usefulness of simple interventions. A single online education session within elite basketball [15] as well as a series of educational videos or online pamphlets for high school athletes [16] both appear to improve MH knowledge. These results are promising and justify the application of similar strategies in other sport settings.

Given the growing interest in MC tracking and calls for concurrent education to be implemented, there is a need to confirm whether educational interventions can assist in promoting buy-in for tracking and proactive management of MH. This study seeks to fill a gap in the literature, where there is a lack of studies that examine the link between MH education and related communication and applied practices (such as MC tracking) and to observe knowledge retention following an education intervention. Therefore, the primary aim of this study is to determine what effect an education intervention has on female athletes’ MH knowledge, their likelihood of communicating with others in the sporting environment about menstrual-related topics, and their perceived importance of MC tracking immediately and three months post-intervention. A secondary aim is to examine whether factors such as age, education level, or HC use influence these outcomes. It was hypothesised that there would be a difference in all MH knowledge-related outcomes, perceived importance of MC tracking, and likelihood to communicate with coaches and medical staff between pre- and post-intervention, but not from post to follow-up.

## 2. Materials and Methods

**Figure 1:**
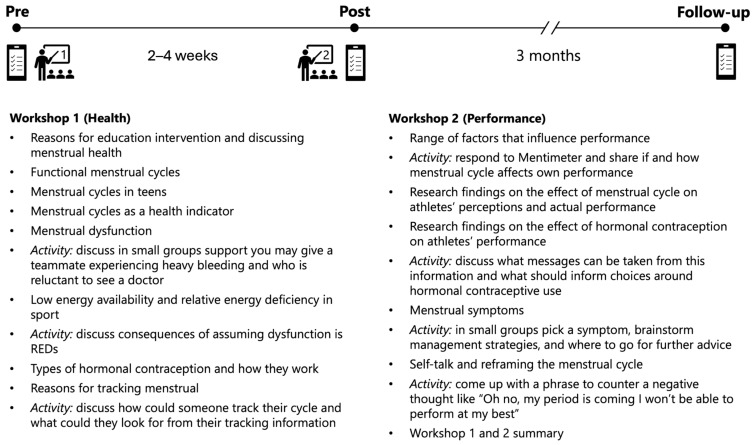
Schematic of study design for the multi-cohort menstrual health education intervention, including the content and activities included in each workshop of the intervention.

A multi-cohort intervention study was conducted to assess MH knowledge, communication, and perceived importance of MC tracking before, after, and three months following a brief education intervention, consisting of two face-to-face workshops (Figure 1) and the provision of take-home resources.

The study was piloted with a professional Australian Football team (Tier 3 [21]), with updates made to information depth, language use, and survey questions. This study received ethical approval from the institutional ethics committee (HEC23438).

### 2.1. Participants

The research team contacted coaches and practitioners working with female athletes and distributed study information via LinkedIn to recruit study cohorts. An eligible cohort was any group of female athletes based in Victoria, Australia, that trained and/or competed together (i.e., team or training squad), with no inclusion criteria based on sport or competitive level. Coaches or practitioners who expressed interest coordinated with researchers to schedule face-to-face workshops and forward the study information to their athletes, who could opt-in to attend the workshops and complete the relevant surveys. Implied consent was obtained by adult participants (i.e., over the age of 18) through their completion of the online surveys, while participants aged 15 to 18 were required to provide their parent/guardian’s written consent prior to undertaking the first survey and participating in the education workshops. The competitive level of the cohort was classified using McKay and colleagues’ [21] framework.

### 2.2. Intervention

Athletes from each cohort attended two 45 min workshops delivered in person by the lead researcher, held between two and four weeks apart to accommodate training schedules. The workshops focussed on health (session 1) and performance (session 2) and included a presentation-style delivery of information and peer engagement through group discussions and interactive activities, as summarised in Figure 1. Additional resources were provided, including handouts summarising types of menstrual dysfunction and an overview of REDs, and laminated posters displaying a flow chart for determining whether an athlete should seek medical support for possible menstrual dysfunction, intended to be displayed within their training environment, such as the changerooms or bathrooms.

### 2.3. Outcomes

Online surveys were housed on QuestionPro [22] and completed at three timepoints: immediately prior to the first workshop, immediately following the second workshop, and three months following the second workshop (Figure 1). At each timepoint, participants completed a MH knowledge assessment consisting of seven true or false questions, eight multiple-choice questions, and two short-answer questions relating to MCs, menstrual dysfunction, and HCs. A combination of questions from assessments from Larsen and colleagues, who assessed MH knowledge in female athletes [16], and elite basketballers and their support staff [15], were selected, with the wording of questions and responses replicated exactly. Additional questions were included following discussion with the authors of an ongoing project validating a MH assessment tool (now published [23]). A MH knowledge score between 0 and 1 was calculated based on the sum of correct responses (i.e., 1 point per true/false and multiple choice question, 1 point per correct response to the short answer question, which each had two correct responses) and divided by 19 (i.e., the maximum possible number of points). The sum of unanswered or “I am not sure” responses divided by 19 created the uncertainty score. At each timepoint, participants used a 10-point Likert scale to rate their perceived MH knowledge, likelihood of discussing MCs, menstrual dysfunction and/or HCs with teammates, coaches, medical staff, and performance staff, and importance of MC tracking. Perceived importance of tracking served as a proxy for buy-in to tracking, as engagement in tracking was not a criterion for inclusion and no information regarding consistency of tracking was collected. Demographic information such as age, gender, HC status, and highest level of education attained were also collected during the first survey. The survey questions and responses are provided in the Supplementary Material.

### 2.4. Statistical Analysis

All survey responses were exported from QuestionPro and compiled into a master spreadsheet in Microsoft Excel (Version 2511, 2025) in a long data format. The responses from athletes that did not attend the initial workshop, provided responses at the pre-intervention timepoint, or completed at least two of the three surveys were excluded. The data were imported to JASP (version 0.19.3.0, 2025), where all statistical analyses were performed. Demographic characteristics are presented as frequencies and percentages for categorical data (i.e., HC use, highest level of education attained) and means and standard deviations for continuous data (i.e., age).

For the primary aim, Bayesian generalised linear mixed models with a beta distribution were produced to understand changes in knowledge and uncertainty scores between timepoints. Knowledge and uncertainty scores were calculated as a proportion of correct or blank/’I am not sure’ responses respectively, based on a possible total of seventeen. To understand changes in ratings of perceived knowledge, importance of tracking, and likelihood to communicate over time, Bayesian linear mixed models were used. All models included the timepoint as a fixed effect and participant as a random intercept. For the secondary aim, differences in all outcomes of interest were then evaluated in relation to the participants’ age (centred around the mean), education level (completed further education following high school or not), and HC use (yes or no). Each of these factors was included in a Bayesian generalised linear mixed model or Bayesian linear mixed model, with participant as a random intercept. Two-way interactions for Timepoint*Age, Timepoint*Education level, and Timepoint*HC use were investigated. Three-way interactions were not analysed due to sample size constraints. Given age was the only continuous factor considered in these two-way interactions, flexplots were visually inspected and where a non-linear relationship between age and an outcome was observed, a squared centred age variable (age^2^) was included in the model. All models applied four Markov Chain Monte Carlo chains of 4000 iterations, following a 2000 iteration warm-up, using a No-U-Turn Sampler (adapt_delta = 0.98) and a maximum tree depth of 15. An R-hat less than 1.01 and an effective sample size greater than 1000 had to be met to confirm convergence and stability of the model. Median posterior estimates and the 95% credible intervals were initially checked to identify a meaningful change over time or influence of age, education, or HC use, and are presented in the results tables. Pairwise contrasts of the estimated marginal means were then specified to identify where credible differences occurred and reported within the results text. Where the pairwise contrasts of the estimated marginal means identify a credible change that the posterior estimates do not, these are still included in the results. In these instances, the posterior median and the 95% highest posterior density (HPD) credible intervals are presented within the results text.

## 3. Results

Three unique cohorts were involved in this study: (1) a multisport group of grade 11 and 12 students at a selective sports high school (Tier 2–3); (2) a women’s soccer team competing in a national competition (Tier 3); and (3) a senior Australian Football (AF) team competing at a local level (Tier 2). The workshops were held at different points in each group’s competition calendar—during school term for the Sport School students who were all at differing points, pre-season for the Tier 3 Soccer team, and in-season for the Tier 2 AF team. The workshops were held at the cohort’s main training facilities, either in lieu of training during the school day, or before or following a field training session. Initially, sixty-eight athletes expressed an interest in participating in this study. Following the removal of those who did not complete the pre-workshop survey and either the post or follow-up survey, the responses of 51 athletes were analysed. The retention of participants was highest in the Selective Sports High School (82%), with retention across all cohorts >60% (Tier 3 Soccer team, 79%; Tier 2 AF team, 61%). The demographic information of the included participants is presented in Table 1.

### 3.1. Effect of the Intervention on Knowledge, Communication, and Importance of Tracking

Overall, the education programme seemed to improve participants’ MH knowledge and uncertainty scores, perceived knowledge, importance of tracking, and likelihood to communicate with teammates and performance staff (Table 2). On average, participants provided three more correct responses, attempted to answer one more question (rather than leaving it blank or selecting ‘I am not sure’), and rated their perceived knowledge one point higher following the intervention. These improvements were retained at follow-up with no credible changes observed between the post-intervention and three-month timepoints.

In terms of communication, participants reported being more likely to discuss menstrual-related topics with teammates and performance staff. These changes in communication were retained three months following the intervention (i.e., no credible change from post-intervention). While there appeared to be a small increase in participants’ likelihood to communicate with medical staff post-intervention, there were no credible changes in communication ratings with medical staff or coaches at any timepoint. There was no change in the rated importance of MC tracking from pre- to post-intervention. However, there was a credible difference from post-intervention to follow-up, in which perceived importance of MC tracking increased at the three-month timepoint.

### 3.2. Influence of Age, Hormonal Contraceptive Use, and Education Level

Age, HC use, and educational level may influence the differences observed in some outcomes between timepoints (Table 3). An interaction effect for timepoint and age was observed on knowledge scores, where it appears that from the post to follow-up timepoints, younger participants’ number of correct responses increased, while older participants demonstrated a small decrease in correct responses. Age may also influence participants’ perceived communication preferences, with older participants increasing their likelihood to communicate with medical staff than younger participants at follow-up compared to post-intervention (median difference = 8.08, 95% HPD [6.88, 9.23]).

An interaction effect of timepoint and HC use was also observed for several outcomes. HC users showed smaller improvements in the number of correct responses to the MH knowledge assessment than non-HC users between pre-intervention and follow-up. HC users also demonstrated a greater increase in reported likelihood to communicate with medical staff than non-HC users from pre to follow-up (median difference = 1.31, 95% HPD [0.24, 2.37]).

Education level appears to mediate the effect of time, with participants who have attained a diploma or higher showing larger increases in the likelihood to communicate with medical staff from post-intervention to follow-up, compared to participants who have only finished Grade 10 or Grade 12 (median difference = 1.33, 95% HPD [0.14, 2.51]). Finally, there was no credible influence of age, education level, or HC use on the effect of the intervention on participants’ ratings of the importance of MC tracking.

## 4. Discussion

An education programme consisting of two 45 min workshops and targeted handouts appears to be an effective way to improve athletes’ MH knowledge and increase their likelihood of discussing MH with teammates and performance staff. The findings of the current study also suggest changes are retained for three months following the intervention, with age, education level, and HC use appearing to impact results to some degree. There was, however, no change in the likelihood to communicate with coaches or medical staff following the intervention observed. A novel aspect of this study was the investigation of athletes’ perceptions of MC tracking, with results showing there may be a delayed increase in the rated importance of MC tracking following the intervention.

The average pre-intervention knowledge score of 46% in this study is similar or slightly better than previously observed athlete groups that have assessed MH knowledge [13,14,15]. Using a similar assessment tool, elite basketballers (Tier 4) [15] and professional soccer players (Tier 3) [13] report knowledge scores of ~40%, while Australian athletes at Tier 3 and above [14] and developmental athletes within professional soccer [13] have reported knowledge scores of 35% and 28% respectively. Earlier MH knowledge assessments from 2020, with lower scores of 35% [13], may suggest a growing awareness of MH in Australian athletes over the last 5 years. This could, in part, be attributed to greater recognition from practitioners that MH is an important aspect of athletes’ health and wellbeing [24] and more conversations of female athlete health and performance being undertaken, with prominent athletes publicly sharing their experiences with MH-related challenges [25]. The relatively low scores in developmental athletes could be the result of their generally younger age and presumably less personal experience or contact with relevant health services and information, which has been seen in soccer athletes [13].

Overall, the average improvement in knowledge over time (pre- to post-intervention) in our study (+18%) was smaller than that observed by Larsen and colleagues [15] (+35%), whose study was an intervention using a single online education session. The smaller improvements observed in our study could be attributed to the design of the intervention, with two workshops being delivered two to four weeks apart, meaning participants needed to retain information presented in the first workshop before completing the knowledge assessment following the second workshop. In addition to an objective knowledge assessment, the present study recorded athletes’ perceived level of knowledge, which appeared to improve following the intervention. Perceived knowledge has been recorded in studies with adolescent and collegiate athletes [26,27,28], similarly showing improvements following face-to-face presentations and discussion sessions. This suggests education may improve athletes’ perceptions of their knowledge (and potentially their confidence). Athletes’ perception and confidence in their knowledge, not just an actual change in knowledge, could impact behaviour similarly. This is an important consideration for future research that examines the effect of MH education interventions on outcomes beyond MH knowledge, such as communication behaviour. The improved perception of knowledge is likely reflected in the finding that the measure of uncertainty decreased following the intervention, as participants attempted more questions (i.e., rather than leaving it blank).

One of the strengths and novel aspects of the present study is the inclusion of a follow-up assessment. Follow-up assessments are rarely used in existing research on MH education interventions for athletes, except for studies focused solely on FT knowledge [18,20]. However, these only compared outcomes from pre to follow-up, not post to follow-up, meaning retention following the education could not be assessed properly [18,20]. A three-month follow-up period was chosen to capture medium-term retention while minimising participant drop-out due to changes in teams or organisational structures that may occur over longer periods of time [29]. An important finding was that actual and perceived knowledge and likelihood to communicate with teammates and performance staff were retained for at least 3 months. To understand decays in knowledge, future research could adopt greater follow-up periods to examine longer-term retention of knowledge and changes in communication behaviour. Practically, refreshers or subsequent sessions could be implemented after three months or subsequent training cycles to ensure athletes remain knowledgeable enough to proactively manage their health and performance; however, this is yet to be explored in MH education research to date. 

Observing athletes’ self-reported likelihood to discuss MH with others in the sporting environment, our study shows the likelihood to communicate with teammates and performance staff improved following education but remained unchanged for coaches and medical staff. Athletes in the present study were already highly likely to discuss MH with medical staff pre-intervention; hence, there was little room for change to be observed. Improved likelihood to discuss MH with teammates but not coaches following an education intervention has also been observed in adolescent endurance athletes [26]. The lack of change in likelihood to communicate with coaches likely points to specific barriers limiting this communication pathway, which cannot be addressed via athlete education alone. The barriers reported in previous research include athletes’ perceptions of their coach’s knowledge or comfort level [30] or trust that coaches will be considerate and empathetic in these discussions [5]. The low preference for athletes to discuss their MH with coaches suggests that a coach’s role could instead focus more on creating supportive and safe environments for athletes. This would include developing processes and structures that ensure athletes have access to appropriately skilled (e.g., physician, dietitian; either as part of their organisation or on a referral basis) as well as culturally safe individuals (e.g., specific to religion or gender) to talk to. Inclusion of coaches in MH education might improve athletes’ perceptions of the coach’s knowledge to address this potential communication barrier. Furthermore, the use of peer-led education interventions or inclusion of peer-support strategies should be considered in sport settings where there may be barriers to communication between athletes and practitioners. Future research comparing the impact of peer-, coach/practitioner- and ‘expert’-led education interventions would also be warranted, given participants in our study demonstrated a greater likelihood of discussing MH with teammates than with coaches or performance staff. This is also warranted as the present study utilised an ‘expert’-led intervention and it is possible communication behaviours are affected by who delivers the education. Comparisons of the effectiveness of education interventions utilising different modes of delivery (e.g., online vs. face-to-face) would also be valuable. 

The present study observed that MH knowledge and the efficacy of education interventions may be influenced by HC use and age but not by education level. HC users could presumably have greater knowledge than non-HC users, as observed in the present study and previous cross-sectional research assessing athletes’ MH knowledge [13,14,31], due to potentially having more contact with the health system to receive a HC prescription. Pre-intervention, MH assessment outcomes did not appear to differ based on age, agreeing with the finding that age was not a significant factor influencing knowledge scores of national and international level (Tier 3) Australian athletes [14]. However, younger athletes in the present study appeared to retain information from the intervention better than older athletes over the three-month follow-up period. Our findings do not suggest education level influences MH knowledge, contradicting previous cross-sectional research reporting that athletes who have completed a higher level of education achieve greater knowledge scores [13,14]. This disparate finding may be due to the make-up of our participant group, where the small sample size limits the interpretability of the findings. Alternatively, as the awareness of MH for athletes improves, the role of sport organisations to deliver this information to their athletes, regardless of age, may reduce the importance of an individual’s education level. Future studies should aim to recruit larger samples and conduct more nuanced modelling to confirm these findings.

Despite our results showing no overall effect of time on communication with medical staff, older athletes were more likely than younger athletes to discuss MH with medical staff following the intervention. Whilst previous research has not necessarily distinguished the type of practitioner, there is evidence to suggest that older athletes are more comfortable discussing MH with coaches [32]. Based on the present and existing research, older athletes may be more receptive to education, leading to them having larger increases in their likelihood to discuss MH with medical staff post-intervention. Our findings also indicate that HC use and education level may influence the impact of MH-specific education on the likelihood to communicate with medical staff. Potentially, the experiences gained via interactions with medical professionals in the process of initiating or changing HC use could make athletes in the present study more receptive to messaging around the importance of help-seeking and communication. In addition to education level, greater increases in likelihood to discuss MH with medical staff could also be associated with age, whereby older participants are more likely to have achieved an education level greater than high school. With greater recognition and recent efforts to promote education in women’s sport, future research should also consider the influence of previous exposure to sport-specific MH education or years of experience at a particular competitive level, as this was not collected in the present study. Additionally, with multiple factors potentially at play (i.e., age, HC use, education level), larger-scale research should investigate their role and interactions between these factors.

Finally, our study demonstrates that athletes generally perceived MC tracking to be more important three months following the end of the intervention, with no apparent influence of age, education level, or HC use. The education intervention may have initiated further personal exploration of the topic or allowed time for athletes to experience MC tracking, such that their perceived importance of the topic increased. This could suggest that deliberate education may positively influence athletes’ buy-in to MC tracking. However, this study is limited as perceived importance is only assumed to relate to buy-in, meaning it is too early for such conclusions to be made. Where education is utilised as a means of promoting athlete buy-in to monitoring practices in high-performance sport settings, given there is a delayed effect, the timing of such education should be considered. Further research on the applicability of education for this purpose is warranted based on this finding and should also consider exploring specific MC tracking behaviour, and not just their perceived importance of tracking.

### 4.1. Limitations

The conclusions of the present study are limited by a generally small sample size, particularly when interpreting the interaction effects of age, HC use, and education level. To better understand the effects of MH education interventions, future research should focus on recruiting larger sample sizes and more diverse cohorts. Wide-ranging recruitment strategies and the inclusion of cohorts from various locations (e.g., states, countries) and sport types could improve the size and diversity of the sample. Aesthetic sports, para-sports, endurance sports, and training squads of individual athletes from sports such as track and field and swimming were not represented in this study. Another limitation of this study is the lack of a control group, which could be utilised to understand whether the face-to-face, interactive nature of the workshop provided in the present study is similarly effective as a basic online or flyer-based intervention. The education intervention and follow-up periods for each cohort occurred at different points within the sporting season, potentially influencing the knowledge improvements and retention (i.e., it is plausible that athletes could be more motivated and less fatigued early in pre-season compared to during their competitive season, improving their ability to absorb new information). While it may be considered a strength to still observe the study findings irrespective of timing, to potentially maximise the impact of education workshops, consideration of when these are implemented within the competitive season should be investigated or controlled for in future research.

### 4.2. Practical Applications

A brief education programme could be a practical and effective strategy to promote MH literacy and athletes’ perceived importance of MC tracking, and potentially improve communication in sport settings. However, the improvements in communication appear to be limited to discussing MH with teammates or performance staff. Practically, a brief interactive education programme could be utilised in amateur to elite sport settings to ensure athletes are aware of the role of MH in overall health and performance. Given athletes seem to be more likely to discuss MH with their teammates and performance staff following education, influential athletes in the training environment could be well-positioned to reinforce the key messages of MH education. Additionally, with education potentially acting as a catalyst for athletes to start MH-related discussions, practitioners could benefit from education in anticipation of athletes initiating conversations. At this stage, with only a small evidence base, practitioners should focus on introducing education for all athletes, rather than tailoring education to athlete characteristics such as HC use or education level, especially given the inconsistent impact on some, but not all, aspects (e.g., communication versus knowledge).

## 5. Conclusions

This cohort study shows encouraging preliminary results regarding the use of MH education interventions in sport settings. A brief education programme consisting of two face-to-face workshops appears to improve athletes’ knowledge of MH-related topics and increase their perceived level of knowledge. Furthermore, athletes report greater perceived importance of MC tracking and being more likely to discuss MH with their teammates and performance staff, but not medical staff and coaches, following this education. Further research is required to confirm these findings and understand the impact of similar education interventions on communication behaviours and engagement in MC tracking.

## Figures and Tables

**Table 1:** Demographic information of participants, presented as frequency (percentage) or mean ± standard deviation.

	Overall (n = 51)	High School (n = 18)	Tier 3 Team (n = 19)	Tier 2 Team (n = 14)
**Age**	23.40 ± 7.15	17.19 ± 0.51	23.09 ± 4.15	31.35 ± 7.05
**HC Use**				
Yes	22 (43.14)	2 (11.11)	9 (47.37)	11 (78.57)
No	29 (56.86)	16 (88.89)	10 (52.63)	3 (21.43)
**Edu Level**				
Grade 10	21 (41.18)	18 (100.00)	3 (15.79)	-
Grade 12	10 (19.61)	-	10 (52.63)	-
Diploma	5 (9.80)	-	2 (10.53)	3 (21.43)
Bachelor’s	8 (15.69)	-	2 (10.53)	6 (42.86)
Honour’s/Master’s	7 (13.73)	-	2 (10.53)	5 (35.71)

Abbreviations: Edu level = highest level of education attained; HC = hormonal contraceptive.

**Table 2:** Menstrual health assessment scores and ratings across the three timepoints. Presented as the Estimated Marginal Means [posterior median and 95% highest posterior density intervals] for each timepoint and the posterior estimates and 95% CIs to understand the effect of timepoint on results.

Outcome	Pre- Intervention	Post- Intervention	Follow-Up	Effect of Timepoint		
				Est	SE	95% CI
**MH Assessment (0** **–** **1)**						
Knowledge	0.46 [0.40, 0.52]	0.64 [0.58, 0.70]	0.58 [0.51, 0.64]	−0.500	0.121	**[−0.739, −0.259]**
Uncertainty	0.28 [0.23, 0.34]	0.20 [0.15, 0.26]	0.18 [0.13, 0.24]	0.422	0.141	**[0.142, 0.694]**
**Ratings (1** **–** **10)**						
Perceived knowledge	4.40 [3.92, 4.90]	5.70 [5.16, 6.22]	5.90 [5.38, 6.40]	−1.148	0.169	**[−1.482, −0.817]**
Importance of tracking	8.03 [7.59, 8.49]	8.09 [7.58, 8.62]	8.94 [8.44, 9.44]	0.598	0.226	**[0.166, 1.049]**
**Communication w/**						
Teammates	5.87 [5.25, 6.47]	7.01 [6.30, 7.71]	7.23 [6.52, 7.87]	−1.018	0.273	[−1.555, −0.480]
Coaches	4.31 [3.45, 5.09]	4.24 [3.34, 5.18]	4.62 [3.80, 5.39]	−0.094	0.376	[−0.814, 0.665]
Medical Staff	7.45 [6.87, 8.02]	7.94 [7.32, 8.58]	7.87 [7.27, 8.40]	−0.374	0.207	[−0.781, 0.039]
Performance Staff	5.29 [4.43, 6.12]	6.81 [5.84, 7.70]	6.45 [5.63, 7.29]	−1.095	0.347	**[−1.775, −0.399]**

Abbreviations: Est = estimate; MH = menstrual health; SE = standard error; w/ = with. Note: Results for MH assessment scores presented in proportions and bold indicates a credible effect.

**Table 3:** The interaction effect of timepoint and hormonal contraceptive use, education level, and age on menstrual health assessment scores and ratings. Presented the posterior estimates and 95% CIs to understand the interactions between timepoint and other factors on results.

Outcome	Timepoint × HC Use			Timepoint × Education			Timepoint × Age		
	Est	SE	95% CI	Est	SE	95% CI	Est	SE	95% CI
**MH Assessment (0** **–** **1)**									
Knowledge	−0.379	0.167	**[−** **0** **.706, −** **0** **.058]**	−0.202	0.175	[−0.545, 0.137]	−0.004	0.002	**[−** **0** **.007, −** **0** **.0002]**
Uncertainty	−0.252	0.208	[−0.15, 0.663]	0.013	0.209	[−0.395, 0.431]	0.001	0.002	[−0.002, 0.005]
**Ratings (1** **–** **10)**									
Perceived knowledge	−0.073	0.243	[−0.548, 0.417]	−0.268	0.250	[−0.761, 0.229]	−0.012	0.024	[−0.059, 0.035]
Importance of tracking	0.143	0.288	[−0.409, 0.700]	−0.079	0.283	[−0.641, 0.471]	−0.003	0.002	[−0.008, 0.001]
**Communication w/**									
Teammates	−0.169	0.397	[−0.953, 0.619]	−0.479	0.397	[−1.246, 0.300]	−0.005	0.003	[−0.011, 0.001]
Coaches	−0.458	0.53	[−01.504, 0.57]	−0.131	0.552	[−1.188, 0.983]	−0.003	0.004	[−0.010, 0.004]
Medical Staff	0.522 †	0.28	[−0.043, 1.055]	−0.660 †	0.306	[−1.244, −0.053]	−0.001	0.002 †	[−0.005, 0.003]
Performance Staff	0.047	0.511	[−0.994, 1.053]	0.101	0.525	[−0.947, 1.153]	0.032	0.061	[−0.087, 0.149]

Abbreviations: Est = estimate; MH = menstrual health; SE = standard error; w/ = with. Note: Results for MH assessment scores presented in proportions and bold indicates credible effect (based on posterior estimates). † indicates where the posterior estimates do not identify a credible change but the pairwise contrasts of the estimated marginal means do (these results are reported in-text where applicable).

## Data Availability

The original contributions presented in this study are included in the article. Further inquiries can be directed to the corresponding author.

## Supplementary Materials

The following supporting information can be downloaded at: https://www.mdpi.com/article/10.3390/sports14070266/s1, File S1: Survey.

## Abbreviations

The following abbreviations are used in this manuscript:

MC	Menstrual cycle
MH	Menstrual health
HC	Hormonal contraceptive
LEA	Low energy availability
REDs	Relative energy deficiency

## References

[1] Kindelan K. USWNT Used Innovative Period Tracking to Help Player Performance at World Cup. https://www.goodmorningamerica.com/wellness/story/uswnt-innovative-period-tracking-player-performance-world-cup-64339368.

[2] Kemp E. (2023). ‘They’re all in sync together’: How the Matildas manage their menstrual health. The Sydney Morning Herald.

[3] Allen M. Laura Philipp: How to Work WITH Your Menstrual Cycle, Not Against It. https://www.tri247.com/triathlon-news/elite/laura-philipp-menstrual-cycle-training-racing.

[4] Carmichael M., Roberts A., Perry K., Clarke A. (2025). Tailoring support to the female athlete: A cross-sectional online survey to observe current practices in high performance sport. Int. J. Sports Sci. Coach..

[5] Carmichael M., Perry K., Roberts A., Clarke A. “No One Seems to Know What We Should Be Doing”: Developing a Framework to Guide the Implementation of Menstrual Cycle Tracking in Applied Settings. Proceedings of the 30th Annual Congress of the European College of Sport Science.

[6] Brown N., Knight C.J. (2021). Understanding female coaches’ and practitioners’ experience and support provision in relation to the menstrual cycle. Int. J. Sports Sci. Coach..

[7] Forsyth J.J., Sams L., Blackett A.D., Ellis N., Abouna M.S. (2022). Menstrual cycle, hormonal contraception and pregnancy in women’s football: Perceptions of players, coaches and managers. Sport Soc..

[8] Badenhorst C.E. (2024). The Menstrual Health Manager (MHM): A Resource to Reduce Discrepancies Between Science and Practice in Sport and Exercise. Sports Med..

[9] Howe O.R. (2024). Ethical Risks of Systematic Menstrual Tracking in Sport. J. Bioethical Inq..

[10] Verhoef S.J., Wielink M.C., Achterberg E.A., Bongers M.Y., Goossens S. (2021). Absence of menstruation in female athletes: Why they do not seek help. BMC Sports Sci. Med. Rehabil..

[11] Kyriazis S.M., Kukuljan S., Turner A.I., van der Pligt P., Ducher G. (2012). Energy deficiency, menstrual disturbances and low bone mass: What do Australian exercising females know about the female athlete triad?. Int. J. Sport Nutr. Exerc. Metab..

[12] Verrier A., Knight C.J., Brown N. (2024). Perceptions of menstrual cycle tracking among elite rugby players. J. Sports Sci..

[13] Anderson R., Rollo I., Randell R.K., Martin D., Twist C., Grazette N., Moss S. (2023). A formative investigation assessing menstrual health literacy in professional women’s football. Sci. Med. Footb..

[14] Larsen B., Morris K., Quinn K., Osborne M., Minahan C. (2020). Practice does not make perfect: A brief view of athletes’ knowledge on the menstrual cycle and oral contraceptives. J. Sci. Med. Sport.

[15] Larsen B., Minahan C., McLay-Cooke R., Cox K., Bird S.B. (2022). A single online education session improves menstrual cycle and hormonal contraceptive knowledge in elite female basketball players and their support staff. N. Z. J. Sports Med..

[16] Roche M., McIntyre A., Oliver C., Sainani K., Boyd T., Stoner A., Kraus E. (2024). How can we better engage female athletes? A novel approach to health and performance education in adolescent athletes. BMJ Open Sport Exerc. Med..

[17] Brown K., Yates M., Meenan M., Brown A.F. (2020). Increased Female Athlete Triad Knowledge Among Collegiate Dancers Following a Brief Educational Video Intervention. J. Danc. Med. Sci..

[18] Stewart T.M., Pollard T., Hildebrandt T., Wesley N.Y., Kilpela L.S., Becker C.B. (2019). The Female Athlete Body project study: 18-month outcomes in eating disorder symptoms and risk factors. Int. J. Eat. Disord..

[19] Krick R.L., Brown A.F., Brown K.N. (2019). Increased Female Athlete Triad Knowledge Following a Brief Video Educational Intervention. J. Nutr. Educ. Behav..

[20] Doyle-Lucas A.F., Davy B.M. (2011). Development and evaluation of an educational intervention program for pre-professional adolescent ballet dancers: Nutrition for optimal performance. J. Danc. Med. Sci..

[21] McKay A.K.A., Stellingwerff T., Smith E.S., Martin D.T., Mujika I., Goosey-Tolfrey V.L., Sheppard J., Burke L.M. (2022). Defining Training and Performance Caliber: A Participant Classification Framework. Int. J. Sports Physiol. Perform..

[22] QuestionPro. https://www.questionpro.com/au.

[23] Larsen B., Greet E., Bird S.P., Quinn K., McNamara A., Osborne J.O. (2025). The Development of a Valid and Reliable Questionnaire to Measure Menstrual Cycle and Hormonal Contraceptive Knowledge Among Athletes and Sports Performance Support Staff. Scand. J. Med. Sci. Sports.

[24] Fortington L.V., Badenhorst M., Bolling C., Derman W., Emery C.A., Pasanen K., Schwellnus M., Verhagen E., Finch AO C.F. (2023). Are we levelling the playing field? A qualitative case study of the awareness, uptake and relevance of the IOC consensus statements in two countries. Br. J. Sports Med..

[25] Trosic J. A Period That Starts a Conversation: Female Athletes Break the Taboo on Menstruation in Elite Sport. https://www.olympics.com/en/news/menstruations-elite-sport-break-taboo-improve-female-athletes-performance-period.

[26] Solli G.S., De Martin Topranin V., Svantorp-Tveiten K.M.E., Noordhof D.A. (2025). The Effect of an Educational Session to Improve Knowledge About the Menstrual Cycle and Hormonal Contraceptives in Junior Endurance Athletes and Their Coaches: An Exploratory Investigation. Int. J. Sports Physiol. Perform..

[27] Skowron P., Kaur R., Cage S., Wadle C., Trail L., Warner L. (2025). Impact of an Education Session on Improving Female Collegiate Athletes’ Comfort with Women’s Health Topics. Internet J. Allied Health Sci. Pract..

[28] Schulz J.M., Hewitt C.M., Stellingwerff T., Stellingwerff H., Ackerman K.E., Thornton J.S. (2025). Mapping the gap: A study on the confidence in knowledge and reporting of relative energy deficiency in sport (REDs) in Canadian University cross-country coaches and athletes. Int. J. Sports Sci. Coach..

[29] Meaney P.A., Joyce C.L., Setlhare S., Smith H.E., Mensinger J.L., Zhang B., Kalenga K., Kloeck D., Kgosiesele T., Jibril H. (2019). Knowledge acquisition and retention following Saving Children’s Lives course for healthcare providers in Botswana: A longitudinal cohort study. BMJ Open.

[30] von Rosen P., Ekenros L., Solli G.S., Sandbakk Ø., Holmberg H.C., Hirschberg A.L., Fridén C. (2022). Offered Support and Knowledge about the Menstrual Cycle in the Athletic Community: A Cross-Sectional Study of 1086 Female Athletes. Int. J. Environ. Res. Public Health.

[31] Majumder T., De Martin Topranin V., Sandbakk Ø., Noordhof D.A. (2022). Indian Endurance Athletes’ Menstrual Cycle: Practices, Knowledge, Communication, Health, and Changes in Perceptions Across the Phases. Int. J. Sports Physiol. Perform..

[32] Srinivasa Gopalan S., Liu S., Mann C., Buckler E.J. (2024). Examining the coach–athlete relationship for facilitators and barriers to healthy sport participation for cyclically menstruating athletes: A systematic review. Int. J. Sports Sci. Coach..

