# Supplementary material for: Effects of a Menstrual Health Education Intervention on Female Athletes’ Knowledge and Communication
Source: Sports (Basel). 2026 Jun 26;14(7):266. doi: 10.3390/sports14070266 (PMC13417738; doi:10.3390/sports14070266)
Supplement: Supplementary file 1 [file sports-14-00266-s001.zip › sports-4306901-supplementary.pdf]

Survey

Demographic Questions

Which of the following best describes you?

- Female
- Male
- Non-binary
- Prefer not to say

What is your date of birth (dd/mm/yyyy)?

What is the highest level of education you have completed?

- Primary school
- Secondary school (grade 10)
- Senior secondary school (grade 12)
- Diploma
- Bachelor's degree
- Honours/Master's degree
- Doctorate

Have you ever used any form of hormonal contraception (e.g. oral contraceptive pill, Mirena, Implanon, etc)

- Yes
- No

Do you currently use any form of hormonal contraception (e.g. oral contraceptive pill, Mirena, Implanon, etc)?

- Yes
- No

MH Knowledge Assessment

Are the following statements true or false?

	True	False	I am not sure
The normal amount of menstrual fluid (blood) to lose during menstruation (i.e., one period) is 100 mL	❑	❑	❑
Menstruation may stop in a female who doesn't consume enough energy to support daily living and training activities	❑	❑	❑
The copper IUD is a hormonal contraceptive option (i.e., contains synthetic hormones)	❑	❑	❑
Persistent absence of menstruation (i.e., 'missing periods') can increase the risk of poor bone health	❑	❑	❑
Having an irregular menstrual cycle is often a sign that female athletes are in peak competitive shape	❑	❑	❑
It is normal to experience debilitating pain that interferes with day-to-day activities prior to or during menstruation	❑	❑	❑
When taken properly, the combined oral contraceptive pill is ≥99% but <100 % effective at preventing pregnancy	❑	❑	❑

For someone with a regular menstrual cycle, the length of one complete menstrual cycle (i.e., the number of days from the first day of one period to the first day of the next period) is:

- 6-20 days
- 21-35 days

36-44 days
I am not sure

At what age do most girls begin to menstruate (i.e., get their first period)?
7-10 years
11-14 years
15-18 years
I am not sure

How many days before starting menstruation (i.e., the first day of a period) does ovulation typically occur?
14 days
7 days
1 day
I am not sure

What is amenorrhea?
a condition of absent menstruation
a condition of infrequent menstruation
a condition of painful menstruation
I am not sure

If someone has tissue similar to the lining of the uterus growing outside of the uterus, they may be diagnosed with:
polycystic ovarian syndrome
secondary amenorrhea
endometriosis
I am not sure

What happens to the natural ovarian hormones produced by the body when you take hormonal contraception (e.g., the pill)?
they increase
they decrease
they stay the same
I am not sure

One type of oral contraception is called the combined pill. This form of contraception contains which synthetic hormone(s)?
progesterone
oestrogen and progesterone
testosterone
I am not sure

Another type of oral contraception is called the mini pill. This form of contraception contains which synthetic hormone(s)?
progesterone
oestrogen and progesterone
testosterone
I am not sure

Name the two female sex hormones produced by the ovaries that fluctuate throughout the menstrual cycle:

Name the two distinct phases that occur within one typical menstrual cycle:

How important do you believe it is for athletes to track their menstrual cycle?

	Not important				Somewhat important				Very important
Importance of tracking	1	2	3	4	5	6	7	8	9	10

How would you rate your knowledge of MH (i.e. menstrual cycles, menstrual dysfunction and hormonal contraception)?

	Very poor				Average				Excellent
Knowledge	1	2	3	4	5	6	7	8	9	10

How likely do you think you are to discuss menstrual cycles, menstrual dysfunction and/or hormonal contraception with the following people?

	Not at all likely				Somewhat likely				Very likely
Teammates	1	2	3	4	5	6	7	8	9	10
Coaches	1	2	3	4	5	6	7	8	9	10
Medical staff	1	2	3	4	5	6	7	8	9	10
Performance staff	1	2	3	4	5	6	7	8	9	10

Open ended question (pre)
Please tell us what you would like to learn more about in relation to menstrual cycles, menstrual dysfunction, hormonal contraceptive and their impact on sports performance.

Open ended questions (post)
What did you find to be most useful about the MH workshops?

What did you learn about yourself in the process of participating in these MH workshops?

How do you plan on applying what you've learned from these workshops as you move forward in your sport?

Do you have any other feedback related to these MH education workshops?

Open ended questions (follow-up)
If so, how did you apply what you learned from these workshops in the last three months?

If you would like to receive a summary of the study findings and a copy of any research outputs, please enter an email address these may be sent to.
